# Does Gastrointestinal Dysmotility Predispose to Recurrent or Severe Forms of *Clostridium difficile* Infections?

**DOI:** 10.1155/2014/575216

**Published:** 2014-12-11

**Authors:** Abdul wahab Hritani, Ahmad Alkaddour, Jeff House

**Affiliations:** University of Florida-Jacksonville, Jacksonville, FL 32209, USA

## Abstract

*Clostridium difficile* infection (CDI) is the most common cause of hospital-acquired diarrhea. A limited number of studies have looked at the risk factors for recurrent CDI. Mitochondrial NeuroGastroIntestinal Encephalopathy (MNGIE) is a rare multisystemic disorder that causes gastrointestinal dysmotility. Herein we present a patient with MNGIE who suffered recurrent and severe *C. difficile* infection despite appropriate treatment. We aim to bring the gastroenterologist's attention to gastrointestinal dysmotility as a possible risk factor for the development of recurrent or severe forms of *C. difficile* infections.

## 1. Introduction

*Clostridium difficile* is a spore-forming Gram-positive anaerobic bacillus that is the most common cause of diarrhea in hospitalized patients [1].* C. difficile* infection (CDI) is the most common cause of hospital-acquired diarrhea [2]. It is a serious medical condition, and although heightened awareness of CDI outbreaks has increased surveillance, the incidence and severity of CDI are increasing around the world. Around 10–40% of patients will experience a posttreatment recurrence of* C. difficile* positive diarrhea in association with a repeatedly positive stool test for* C. difficile* toxin [3]. A limited number of studies have looked at the risk factors for recurrent CDI.

There is limited evidence that gastrointestinal dysmotility may predispose to the recurrence of CDI. There are a myriad of neurological disorders that entail gastrointestinal dysmotility. Here we present a patient with Mitochondrial NeuroGastroIntestinal Encephalopathy which is a rare multisystemic autosomal recessive disorder. Clinical appearance of MNGIE can be heterogeneous, but it is usually initiated by symptoms of gastrointestinal dysmotility, such as diarrhea, vomiting, and pseudoobstruction [4, 5]. It is also characterized by ptosis; progressive external ophthalmoplegia; peripheral neuropathy; and leukoencephalopathy [6].

## 2. Case Description

A 49-year-old Caucasian male with a PMH of Mitochondrial NeuroGastroIntestinal Encephalopathy (MNGIE), coronary artery disease, spina bifida, and gastroparesis presented with weakness, progressive diarrhea, up to 15–20 bowel movements a day, nausea, and vomiting. He denied subjective fevers or abdominal pain.

The patient was diagnosed a year and a half prior to presentation when he presented with 20 years history of ophthalmopathy, 1 year history of polyneuropathy, 20 pounds weight loss, diarrhea, nausea, vomiting, and abdominal pain. His diagnosis was confirmed by genetic testing, and his variant was found to be TYMP c.1142 T > G (p.Leu381Arg).

Physical exam showed tachycardia with soft, nontender abdomen with J tube in site and lower extremities weakness (3/5) as well. Labs showed leukocytosis and positive* C. difficile* toxin antigen detected in stool.

Patient had finished a 14-day course of PO vancomycin for* C. difficile* colitis that he developed while being treated for aspiration pneumonitis with PO clindamycin. He completed the course one week prior to admission. He had also been started on erythromycin for gastroparesis.

During the current admission patient was treated with IV flagyl and PO vancomycin. He started to improve several days afterwards with less bowel movements, only to worsen again afterwards. CT scan of the abdomen was then performed and showed acute colitis with accompanying appendicitis. Patient was then transferred to general surgery where he was treated with PO vancomycin, IV flagyl, and PR vancomycin, which helped improve his symptoms. Patient was eventually discharged on PO vancomycin taper for 4 weeks.

About 5 months later, the patient was readmitted to the hospital with HCAP. He was treated with several antibiotics and developed a recurrence of* C. difficile* which was treated with IV flagyl and PO vancomycin for 14 days, after which the* C. difficile* infection resolved.

Patient had progressive worsening of his condition for which he remained in the hospital for 8 months and passed away eventually after having a very complicated course.

## 3. Discussion

The progressive GI dysmotility of MNGIE is caused by the combined effect of neuromuscular dysfunction and autonomic dysfunction. It occurs virtually in all individuals with MNGIE. Symptoms usually progress over several decades and can affect any part of the GI tract. Gastric and small bowel hypomotility are most common. Symptoms include early satiety, nausea, dysphagia, GERD, episodic abdominal distension, and diarrhea [6].

Studies in animal models suggested that intestinal motility served as a natural defense mechanism against enteric infections by promoting intestinal clearance of the infecting enteropathogen [7–9].

In a recently published meta-analysis of the risk factors for recurrent CDI, continued use of antibiotics after diagnosis of CDI, use of antacid agents, and older age were identified as prognostic risk factors for recurrent CDI [2]. The use of antimotility agents in the treatment of infective diarrhea is discouraged as patients may deteriorate and develop colonic distension [10].

In a recently published review article that looked at 55 patients with CDI treated with antimotility agents, 17 (31%) of these patients clinically deteriorated and developed complications of toxic megacolon or colonic dilatation [10].

Of note, none of these 17 patients who experienced complications of CDI received appropriate antibiotics therapy at the time of the initiation of antimotility treatment.

Our patient experienced a severe form of CDI even though he was initially on appropriate antibiotic therapy. We hypothesize that the GI hypomotility/dysmotility caused by his condition, MNGIE, might have exacerbated his infection or have contributed to the noneradication of the* C. difficile* spores. Although no direct association could be extrapolated, further studies regarding this matter still need to be done.
